# Containing the Transmission of COVID-19: A Modeling Study in 160 Countries

**DOI:** 10.3389/fmed.2021.701836

**Published:** 2021-08-18

**Authors:** Yan Niu, Jia Rui, Qiupeng Wang, Wei Zhang, Zhiwei Chen, Fang Xie, Zeyu Zhao, Shengnan Lin, Yuanzhao Zhu, Yao Wang, Jingwen Xu, Xingchun Liu, Meng Yang, Wei Zheng, Kaixin Chen, Yilan Xia, Lijuan Xu, Shi Zhang, Rongrong Ji, Taisong Jin, Yong Chen, Benhua Zhao, Yanhua Su, Tie Song, Tianmu Chen, Guoqing Hu

**Affiliations:** ^1^Chinese Center for Disease Control and Prevention, Beijing, China; ^2^State Key Laboratory of Molecular Vaccinology and Molecular Diagnostics, School of Public Health, Xiamen University, Xiamen, China; ^3^School of Journalism and Communication, Peking University, Beijing, China; ^4^Media Analytics and Computing Lab, Department of Artificial Intelligence, School of Informatics, Xiamen University, Xiamen, China; ^5^Department of Stomatology, School of Medicine, Xiamen University, Xiamen, China; ^6^Guangdong Provincial Center for Disease Control and Prevention, Guangzhou, China; ^7^Department of Epidemiology and Health Statistics, Xiangya School of Public Health, Central South University, Changsha, China

**Keywords:** COVID-19, transmissibility, mathematical model, the effective reproduction number, epidemic

## Abstract

**Background:** It is much valuable to evaluate the comparative effectiveness of the coronavirus disease 2019 (COVID-19) prevention and control in the non-pharmacological intervention phase of the pandemic across countries and identify useful experiences that could be generalized worldwide.

**Methods:** In this study, we developed a susceptible–exposure–infectious–asymptomatic–removed (SEIAR) model to fit the daily reported COVID-19 cases in 160 countries. The time-varying reproduction number (*R*_*t*_) that was estimated through fitting the mathematical model was adopted to quantify the transmissibility. We defined a synthetic index (*I*_*AC*_) based on the value of *R*_*t*_ to reflect the national capability to control COVID-19.

**Results:** The goodness-of-fit tests showed that the SEIAR model fitted the data of the 160 countries well. At the beginning of the epidemic, the values of *R*_*t*_ of countries in the European region were generally higher than those in other regions. Among the 160 countries included in the study, all European countries had the ability to control the COVID-19 epidemic. The Western Pacific Region did best in continuous control of the epidemic, with a total of 73.76% of countries that can continuously control the COVID-19 epidemic, while only 43.63% of the countries in the European Region continuously controlled the epidemic, followed by the Region of Americas with 52.53% of countries, the Southeast Asian Region with 48% of countries, the African Region with 46.81% of countries, and the Eastern Mediterranean Region with 40.48% of countries.

**Conclusion:** Large variations in controlling the COVID-19 epidemic existed across countries. The world could benefit from the experience of some countries that demonstrated the highest containment capabilities.

## Introduction

Coronavirus disease 2019 (COVID-19) is a novel infectious disease caused by severe acute respiratory syndrome coronavirus 2 virus (SARS-CoV-2) (1). On March 11, 2020, the World Health Organization (WHO) declared a global pandemic of COVID-19 (2). The COVID-19 pandemic has brought tremendous economic and medical burden, and has severely affected people's normal work and daily lives. In most countries, the health system faces collapse, and there are medical shortages of varying degrees (3). In countries affected by COVID-19, many factories and shops have been closed, and thus many people have lost their jobs and income. The International Labor Organization estimated that 25 million jobs were lost, which could lead to a loss of at least US$220 billion in income in developing countries (4). In 2020, the World Travel and Tourism Council warned that up to 1.67% of the world's gross domestic product could be lost due to the reduction in flight travel (5). Therefore, it is very important for the world to evaluate the comparative effectiveness of COVID-19 prevention and control across countries.

Current research on COVID-19 mainly focuses on issues such as transmission mechanisms, pathogenic mechanisms, prevention and control measures, and infection control (6). Mathematical models of pandemics are useful for understanding the early transmission dynamics of infections, achieving pandemic prediction, and evaluating the effectiveness of control measures. They have been widely used in the prediction of the basic reproduction number (*R*_0_) of COVID-19, and reports of *R*_0_ for COVID-19 have ranged from 2.6 to 4.71 (6–12). One recent study suggested that the median *R*_0_ of COVID-19 may be up to 5.7 (13), which was much higher than the previously estimated maximum value of *R*_0_. The reported *R*_0_ varied across countries. The extent each country to control the epidemic of COVID-19 in the early stage significantly affect the future development of the global pandemic. Several models, such as the stochastic transmission model (14), and ordinary differential equation model (11, 12, 15, 16), have been used in the research on COVID-19; however, research has yet to be conducted at the global level.

This study developed an innovative mathematical model to assess the transmissibility and control capability of COVID-19 epidemic of 160 countries in the initial phase of global pandemic (before August 22, 2020). Considering that COVID-19 can cause a lot asymptomatic infections (A), we added “A” to the SEIR model and formulated a susceptible–exposure–infectious–asymptomatic–removed (SEIAR) model, which better characterized the transmission of COVID-19 compared to the SEIR model. Reproduction number was used to reflect the transmissibility of COVID-19. We also evaluated the ability of 160 countries to control the COVID-19 epidemic.

## Methods

### Study Design

**Figure 1:**
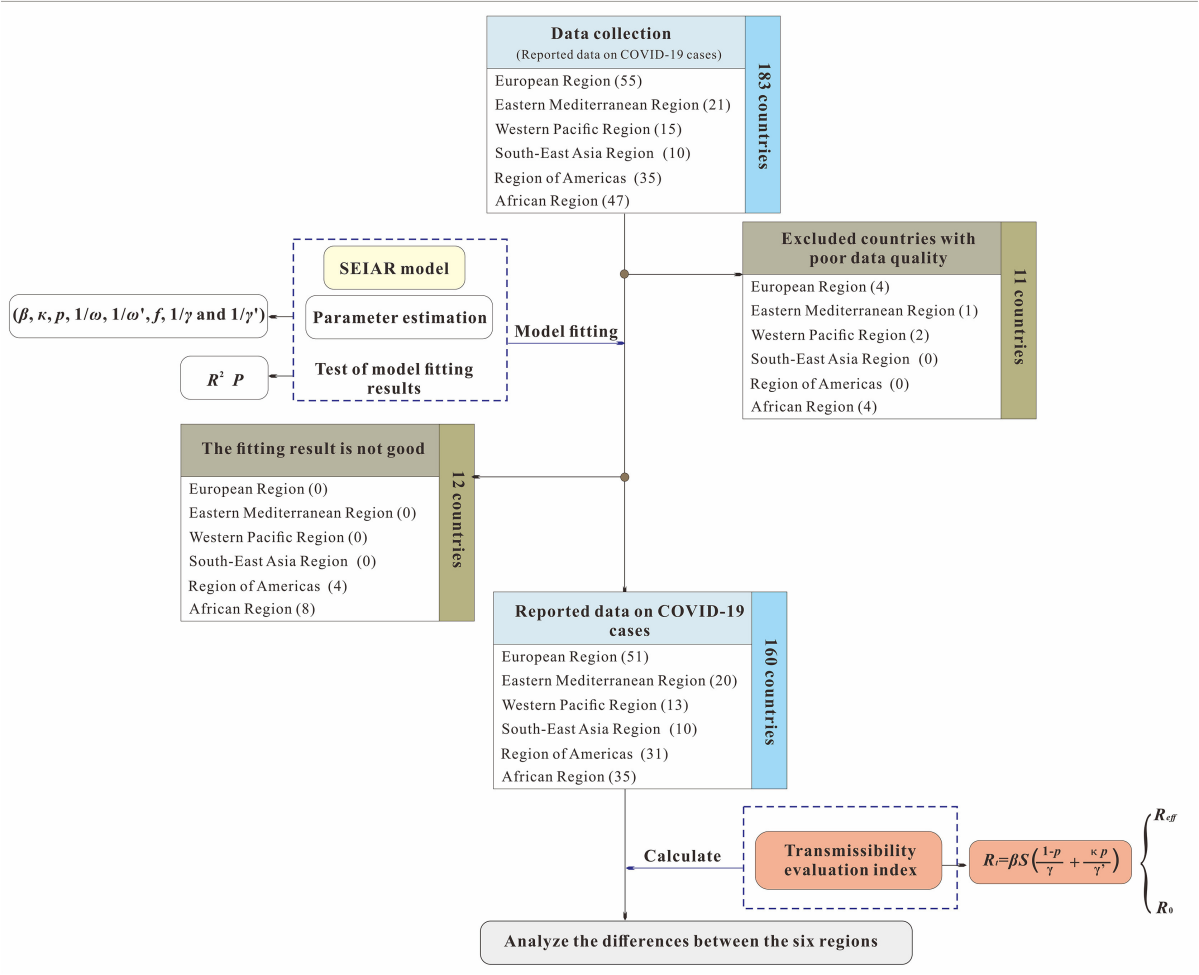
Study design for analyzing the differences of capability to control COVID-19 among 160 countries.

The analysis process used in this study is shown in Figure 1. First, we collected COVID-19 data from each country in the six regions defined by the World Health Organization (WHO) from January 22, 2020, to August 22, 2020, and excluded countries (11 countries) with poor data quality. Second, we fit the mathematical model for the remaining 172 countries and excluded countries (12 countries) due to the poor goodness of fit (the *p* values of goodness of fit was <0.05). Finally, we calculated the transmissibility of COVID-19 and evaluated the prevention and control capacities of the remaining 160 countries.

The level of data monitoring varies across the six WHO regions. The African countries included in the study accounted for 74.47% of the total number of countries in the African region due to insufficient monitoring systems and economic backwardness (17). The remaining five regions were the Southeast Asian Region (100%), the Eastern Mediterranean Region (95.24%), the European Region (92.72%), the Region of Americas (88.57%), and the Western Pacific Region (86.67%). The three countries with the highest cumulative COVID-19 cases were the United States (5,667,112), Brazil (3,582,362), and India (3,044,940). Although some countries in the Western Pacific region, such as China and South Korea, were reported earlier among the countries with the highest cumulative total, the increase of number of cases slowed in the later period. The cumulative number of COVID-19 cases in the European region was between 50,000 and 1,000,000.

### Data Sources

To determine the newly confirmed cases and cumulative deaths globally, we used data from the repository published by Johns Hopkins University Center for Systems Science and Engineering and the Esri Living Atlas team and Johns Hopkins University Applied Physics Lab. Then, we applied our codes to clean and process off-the-shelf data for model building. Our code repository will be published after the paper is accepted.

### The Model of COVID-19

**Figure 2:**
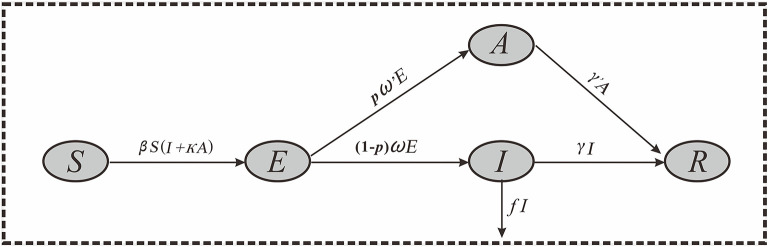
Flowchart of the SEIAR model.

Considering that COVID-19 can cause asymptomatic infections, we developed a SEIAR model based on our previous work (15, 18). The SEIAR model involves five group of populations: susceptible individuals (S), individuals in incubation period (E), symptomatic individuals (I), asymptomatic individuals (A), and recovered individuals (R). A SEIAR model was developed based on the reported data of COVID-19. The model framework is shown in Figure 2.

The model was based on the following assumptions:

1) Assuming that the infection rate coefficient after effective contact between *S* and *I* is β, and assuming that *A* is infectious, and the transmissibility is κ (0 < κ < 1) times that of *I*, then at time *t*, the number of new infections is β*S* (*I* + κ*A*).
2) Assuming that the proportion of asymptomatic infection is *p*, and the incubation period and latent period are 1/ω and 1/ω′, respectively. At time *t*, the number of people who change from *E* to *A* and I are *p*ω′*E* and (1–*p*) ω*E*, respectively.
3) Assuming that the time interval from the onset of the case *I* to the first diagnosis is 1/γ, then at time *t*, the number of people who change from *I* to *R* is γ*I*. Because there are deaths in the reported data, we set *f* as the case fatality rate, and then the number of deaths at time t is *fI*.
4) Assuming that the infectious period of the recessive infected person *A* is 1/γ′, then at time *t*, the number of people who change from *A* to *R* is γ′*A*.

Based on these assumptions, the equations of the SEIAR model are presented as follows:

$$\begin{array}{lll} \frac{dS}{dt} & = & -\beta S\left(I+\kappa A\right)\\ \frac{dE}{dt} & = & \beta S\left(I+\kappa A\right)-p\omega^{\prime }E-\left(1-p\right)\omega E\\ \frac{dI}{dt} & = & \left(1-p\right)\omega E-\gamma I-fI\\ \frac{dA}{dt} & = & p\omega^{\prime }E-\gamma^{\prime }A\\ \frac{dR}{dt} & = & \gamma I+\gamma^{\prime }A \end{array}$$

### Parameter Estimation

The values of the model parameters and their methods are listed in Table 1. There were 8 parameters in the model: β, κ, *p*, 1/ω, 1/ω′, *f*, 1/γ, and 1/γ′.

**Table 1:** Parameter estimation.

**Parameter**	**Description**	**Value**	**Range**	**Source**
β	Transmission relative rate	-	≥0	Curve fitting
κ	Relative transmissibility rate of asymptomatic to symptomatic individuals	1	0–1	(15)
*p*	Proportion of the asymptomatic	0.2	0–1	(11)
1*/ω*	Incubation of symptomatic	0.2	0–1	(16, 17)
1*/ω′*	Incubation of asymptomatic	0.3333	0–1	(16, 17)
*f*	Fatality of the disease	-	0–1	Reported data calculation
1*/γ*	Recovery rate of symptomatic	0.2	0–1	(12)
1*/γ′*	Recovery rate of asymptomatic	0.1	0–1	(12)

1) The collected data of reported COVID-19 cases were employed to fit the SEIAR model to calculate β in each region. In this study, the nodes were segmented according to the inflection point of the epidemic curve shown by the date of onset, and the models were fitted to estimate the β values at different time periods, namely, β_1_, β_2_,…,β_n_.
2) We assumed that the transmissibility of asymptomatic infection was one times that of symptomatic infection (κ = 1) from information in a previous study (19).
3) Because free-access data of asymptomatic infections were unavailable for each country, we determined the parameter *p* based on the published literatures. Studies showed that the values of *p* ranged from 16.1 to 50.6% (20, 21). In this study, we set a mean value of 0.2 in the model. We also performed a sensitivity analysis with the *p* value of 0.1, 0.2, …, and 0.9, respectively, to assess the uncertainty of our results related to with the parameter *p*.
4) Based on previous studies (22, 23), we set the incubation period of COVID-19 to 5 days, and the latent period to 3 days in this study; therefore, 1/ω = 0.2, 1/ω′ = 0.3333.
5) Parameter *f* was the fatality rate of COVID-19 that was calculated using actual data.
6) Based on a previous study (12), we set the infectious period of symptomatic infection as 5 days and the infectious period of asymptomatic infection as 10 days; therefore, 1/γ = 0.2 and 1/γ′ = 0.1.

### Transmissibility Evaluation Index

In this study, the population was not completely susceptible and artificially adopted some prevention and control measures, so we chose the time-varying reproduction number (*R*_*t*_) to calculate transmissibility. The calculation formula was as follows:

$$\begin{array}{l} R_{t}=\beta S\left(\frac{1-p}{\gamma }+\frac{\kappa p}{\gamma^{{}^{\prime }}}\right) \end{array}$$

The *R*_*t*_ value at the beginning of the epidemic is defined as the basic reproduction number (*R*_0_), which was the initial *R*_*t*_ value of each country. When countries have adopted control measures for COVID-19, *R*_*t*_ was defined as the effective reproduction number (*R*_*eff*_).

A synthetic index (*I*_*AC*_) was constructed to evaluate the capability to control COVID-19 in each country. The calculation formula was as follows:

$$\begin{array}{l} I_{AC}=I_{Tn}+I_{WNi}+I_{Cj} \end{array}$$

In the equation, *I*_*Tn*_ was defined as the grade of time it took to make COVID-19 under control; *n* represented the level of the time required to control the COVID-19 epidemic. We divided the time into five grades (≤ 30, 31–60, 61–90, 91–120, 121–150, and 151–180 days) and gave a value of *n* = 1, 2, 3, 4, 5, and 6, respectively. The specific values are shown in Table 2.

**Table 2:** The specific values of *I*_*Tn*_, *I*_*WNi*_, and *I*_*Cj*_.

	***N***	***I_***Tn***_***
The grade of time it will take to bring COVID-19 under control	1	6
	2	5
	3	4
	4	3
	5	2
	6	1
The grade of wave number of COVID-19	***i***	***I**_***WNi***_*
	1	4
	2	3
	3	2
	4	1
The grade of countries where COVID-19 has been brought under control as of August 22	***j***	***I**_***Cj***_*
	0	0
	1	5

*I*_*WNi*_ is the grade of wave number of COVID-19; *i* represents the number of COVID-19 epidemic fluctuation in each country. When the number of fluctuation is 1, *i* = 1. When the number is 2, 3, and 4, the value of *i* is 2, 3, and 4 respectively. The specific values are shown in Table 2.

*I*_*Cj*_ is the grade of countries where COVID-19 has been brought under control (*R*_*eff*_ <1) as of August 22; *j* represents whether the country under study has the ability to control the COVID-19 epidemic. The condition of whether it can be controlled is whether the country can reduce *R*_*eff*_ to below 1. When the country is able to control COVID-19, *j* = 1, and when it is unable to control it, *j* = 0. The specific values are shown in Table 2.

### Simulation Methods and Statistical Analysis

Berkeley Madonna 8.3.18 software (developed by Robert Macey and George Oster of the University of California, Berkeley at Berkeley. Copyright©1993-2001 Robert I. Macey & George F. Oster) was used for the curve fitting. The fourth-order Runge–Kutta method, with tolerance set at 0.001, was used to perform the curve fitting. The coefficient of determination (*R*^2^) was used to assess the goodness of fit. SPSS 13.0 software (IBM Corp., Armonk, NY, USA) was used to calculate the *R*^2^.

## Results

### Epidemiological Characteristics of the COVID-19 Pandemic of 184 Countries

**Figure 3:**
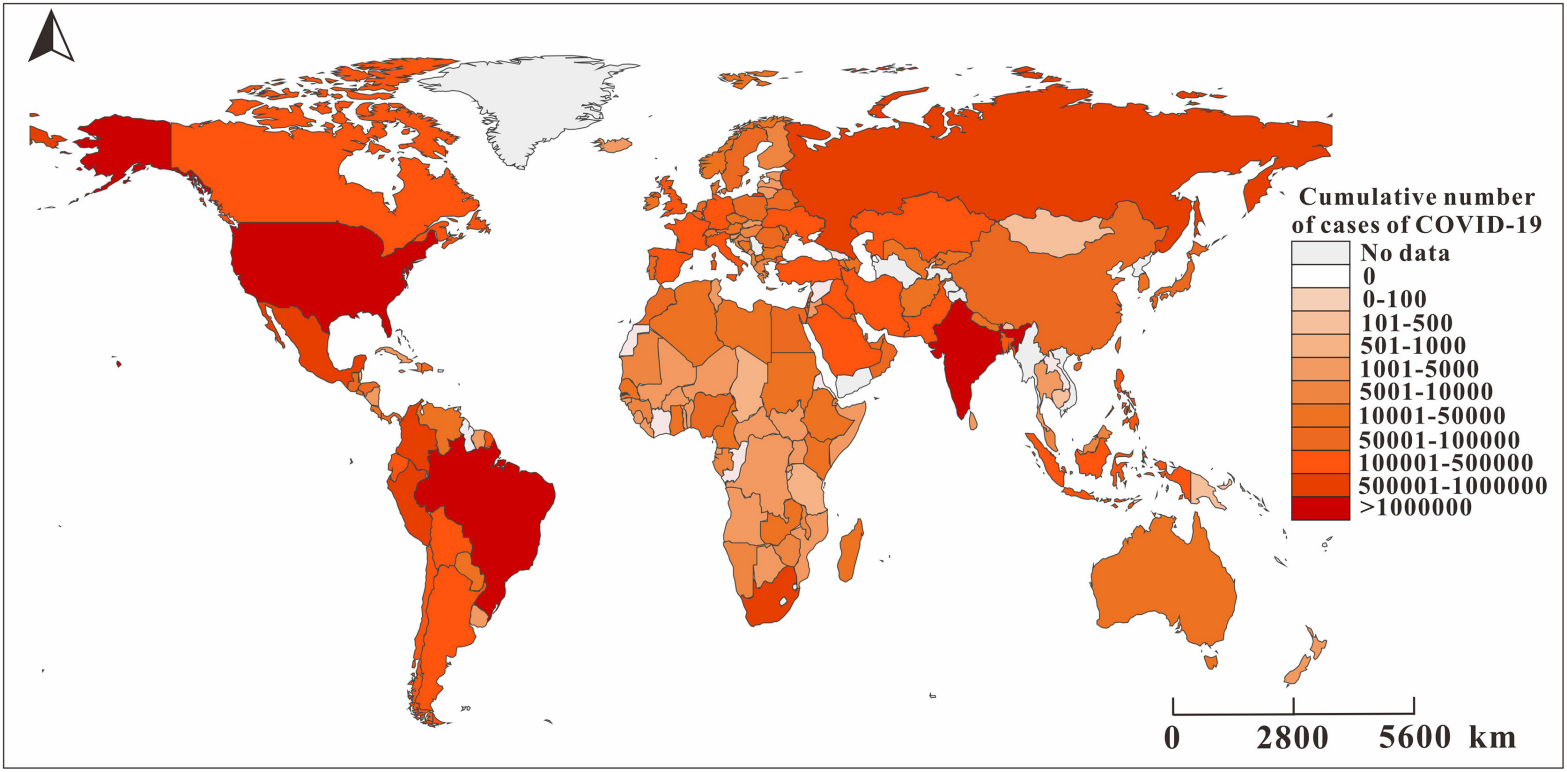
The reported cumulative number of cases of COVID-19 in 160 countries as of August 22, 2020. The map depicted in these figures were taken from Wikimedia Commons (http://commons.wikimedia.org/wiki/Main_Page).

Figure 3 shows the spatial and temporal distribution of the cumulative incidence of COVID-19 in 184 countries since January 31, 2020. By 24:00 on January 31, 2020, the cumulative number of COVID-19 cases in China exceeded 10,000, and there were only a few confirmed cases in other countries. On February 29, the cumulative number of confirmed cases in China exceeded 70,000, and more than 10 cases occurred in some countries in the Western Pacific, Europe, and Canada in the Americas, of which the number of cases in Italy and South Korea exceeded 500 and 3,000, respectively. By March 11, the pandemic had spread globally. The cumulative number of confirmed cases in Italy, France, Spain, Germany, Iran, and other places exceeded 1,000, while the growing rate of China slowed down at this time. By March 31, the COVID-19 pandemic became very serious. The cumulative number of confirmed cases in the United States and Italy exceeded 100,000 cases. By April 21, the cumulative number of confirmed cases in the United States exceeded 800,000, and the cumulative number of newly confirmed cases in France, Spain, Germany, and the United Kingdom exceeded 100,000.

### Model Fitting

**Figure 4:**
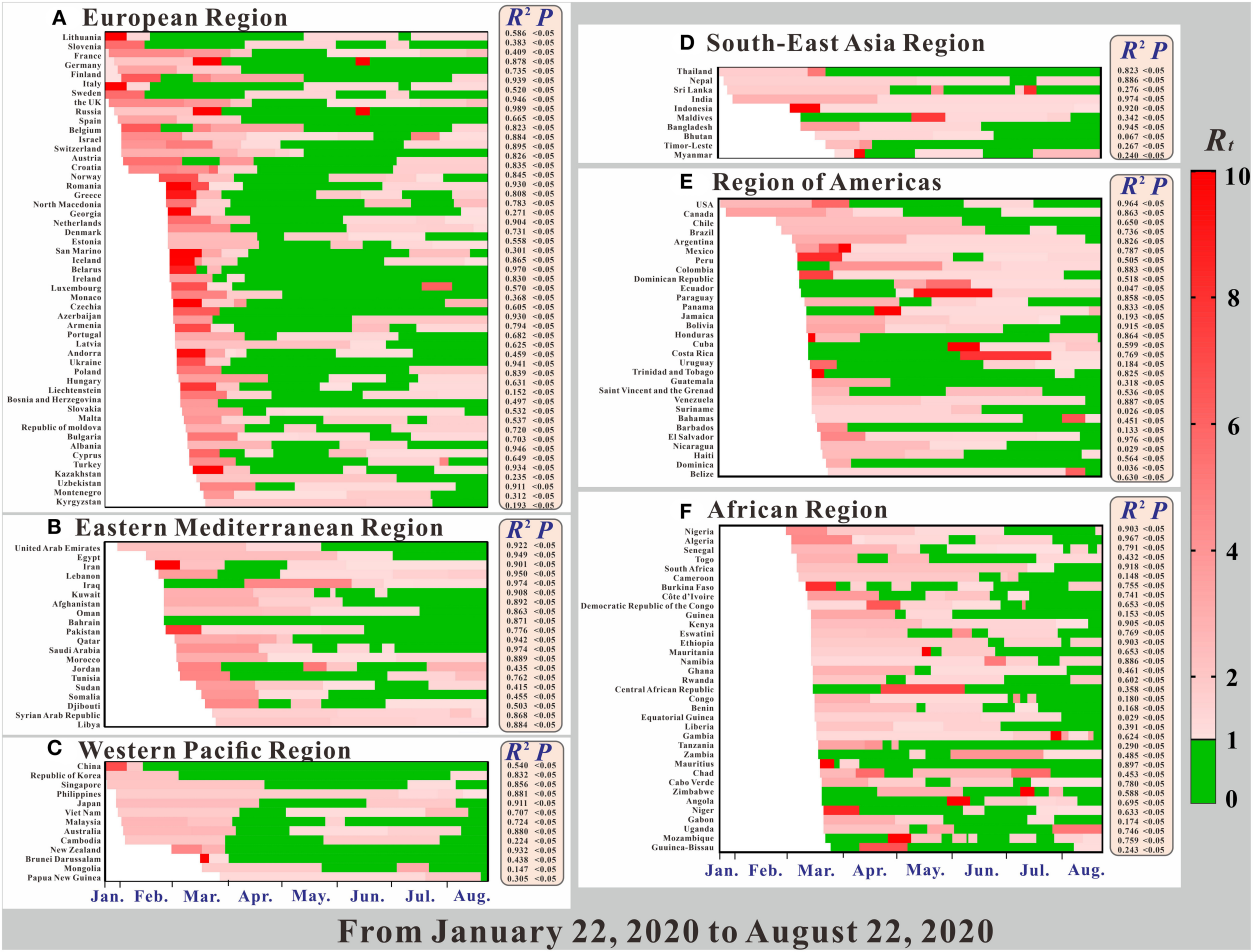
The time-varying reproduction number (*R*_*t*_), the coefficient of determination (*R*^2^), and *p* of model fitting results.

The goodness-of-fit tests showed that the SEIAR model fitted well for the COVID-19 data of most countries (Figure 4). The *R*^2^, which is determined by the number of confirmed cases and the number of fitted new cases, was >0.5 for 112 countries, *p* < 0.05. The *R*^2^ of the Southeast Asian Region, Eastern Mediterranean Region, and European Region were much higher than those of the other regions. However, the SEIAR model did not fit the data of 12 countries. Thus, we excluded the 12 countries from further data analyses.

### Transmissibility of the COVID-19 Pandemic of 160 Countries

**Figure 5:**
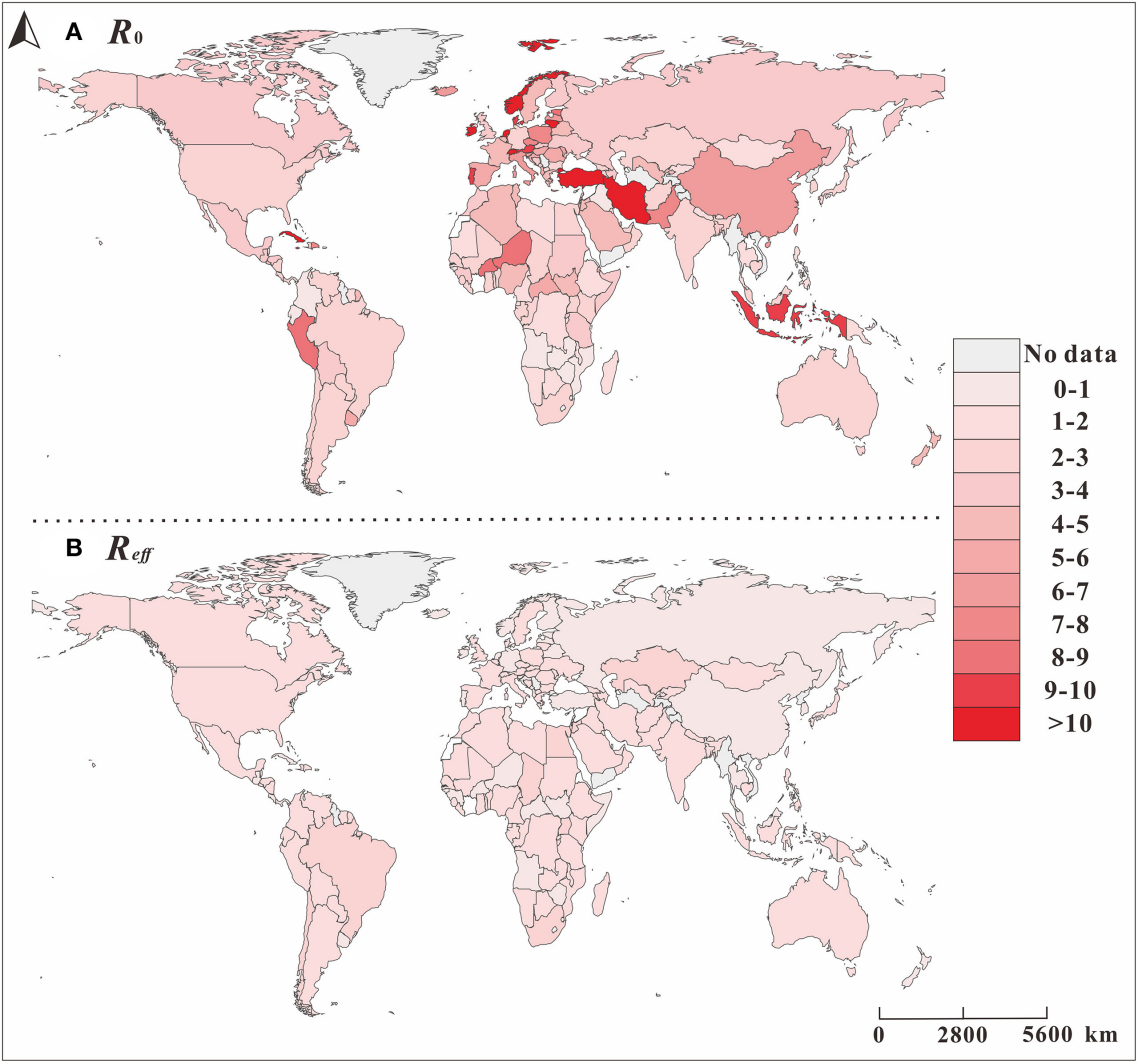
The spatial distribution of basic reproduction number (*R*_0_) and the effective reproduction number (*R*_*eff*_) of COVID-19 in different countries. The map depicted in these figures were taken from Wikimedia Commons (http://commons.wikimedia.org/wiki/Main_Page).

As shown in Figure 5, the *R*_0_ in the early stage of the pandemic was clearly higher than the *R*_*eff*_ value after the control was carried out in the later period, and the *R*_*eff*_ value in a few countries increased in the later period. As shown in Figure 4A, in the early stage of the pandemic, the European Region had the strongest transmissibility of COVID-19, where the *R*_0_ of many countries exceeds 10.

Figure 4 shows a clear relationship between the reproduction number (*R*_*t*_), *R*^2^, and *P* in 160 countries in the six regions. All regions, except for the African region, began to spread COVID-19 on January 22. Among them, the largest *R*_*t*_ range were in the Western Pacific Region and the Region of Americas, and for each, the maximum value was more than 20. Comparted with other regions, the *R*_*t*_ value in the Region of Americas was larger. Secondly, the *R*_*t*_ ranges of the European Region and the African Region were largest, followed by the Eastern Mediterranean Region. The region with the smallest *R*_*t*_ range was the Southeast Asian Region.

Overall, the *R*_*t*_ value of COVID-19 in most countries showed a trend from strong to weak (e.g., China in the Western Pacific Region), but there are also some countries for which the *R*_*t*_ value increased in the later period (e.g., France in the European Region). However, there are still some countries showing further upward trends, such as Sweden, which implemented the policy of “herd immunity”. Of all the regions, changes in the *R*_*t*_ of various countries in the African region are more complicated, and the changes in the *R*_*t*_ values of the other regions are obviously greater. In the Region of Americas, the majority of countries' *R*_*t*_ at the beginning of the pandemic were concentrated and were above 2.0. In terms of overall changes in *R*_*t*_ trends, prevention and control measures were not as effective in the two regions, compared with the other regions. In these two regions, COVID-19 was not effectively controlled in the early stage, making them epicenters of the COVID-19 pandemic today.

### Ability to Control COVID-19 Epidemic of 160 Countries

**Figure 6:**
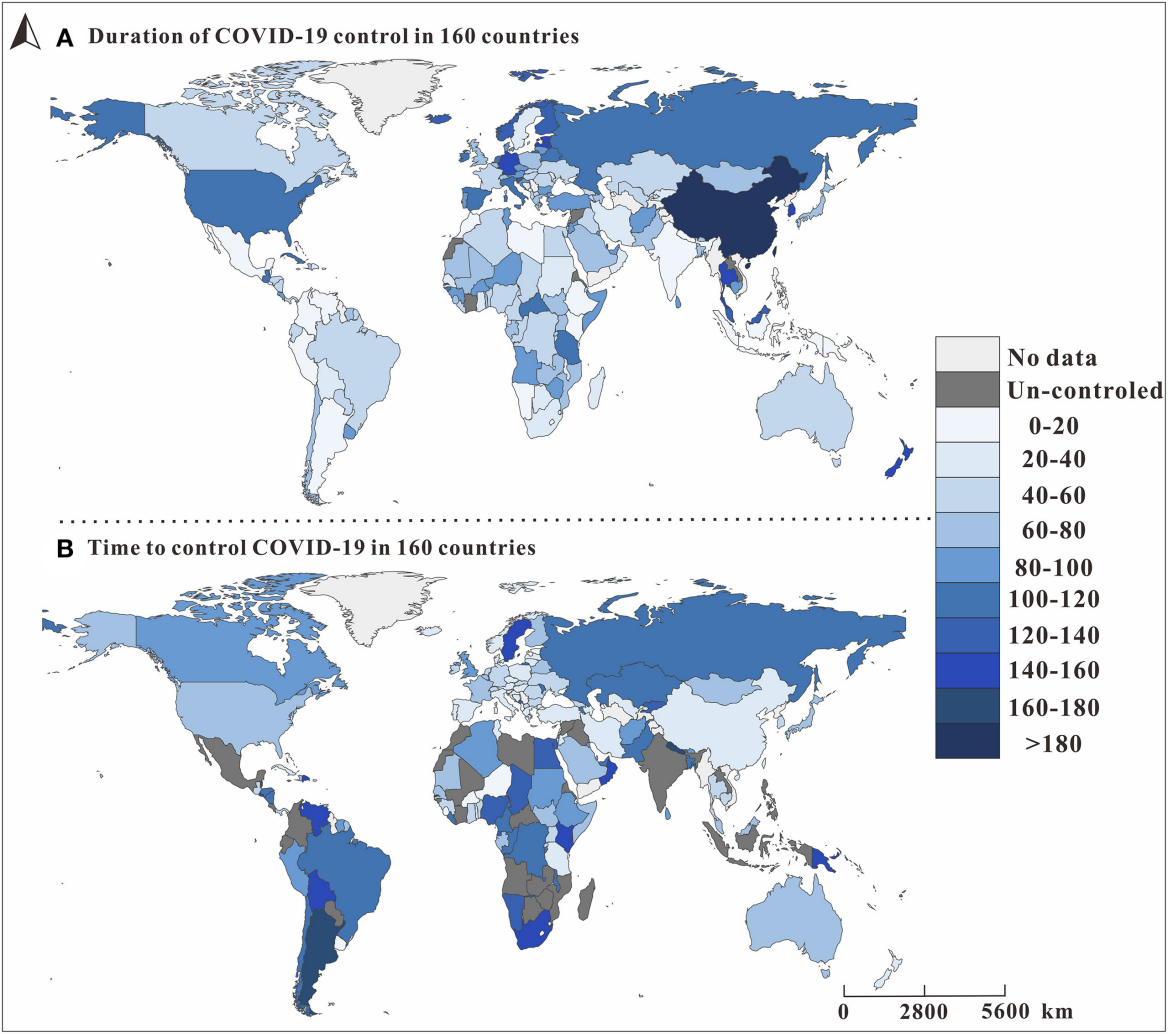
The spatial distribution of *I*_*Tn*_ and *I*_*Cj*_ in 160 countries. *I*_*Tn*_ is defined as the grade of time it will take to bring COVID-19 under control, and *n* represents the level of the time required to control the COVID-19 epidemic. *I*_*Cj*_ is the grade of countries where COVID-19 has been brought under control (*R*_*eff*_ <1) as of August, 22, 2020; *j* represents whether the country under study has the ability to control the COVID-19 epidemic. The map depicted in these figures were taken from Wikimedia Commons (http://commons.wikimedia.org/wiki/Main_Page).

Figure 6A shows that from January 22, 2020, to August 11, 2020, there was great variation in the number of days in the period in which *R*_*t*_ of COVID-19 was <1 among the 160 countries. Compared with Figure 6B, the longer the period in which *R*_*t*_ was 1, the shorter the time needed to control COVID-19. For example, in China in the Western Pacific region, where *R*_*t*_ was <1 for more than 180 days, it took <20 days to reduce *R*_0_ below 1. At the time of the study, many countries in the African Region were not under control, and it took a relatively long time to control COVID-19 in the Regions of Americas.

**Figure 7:**
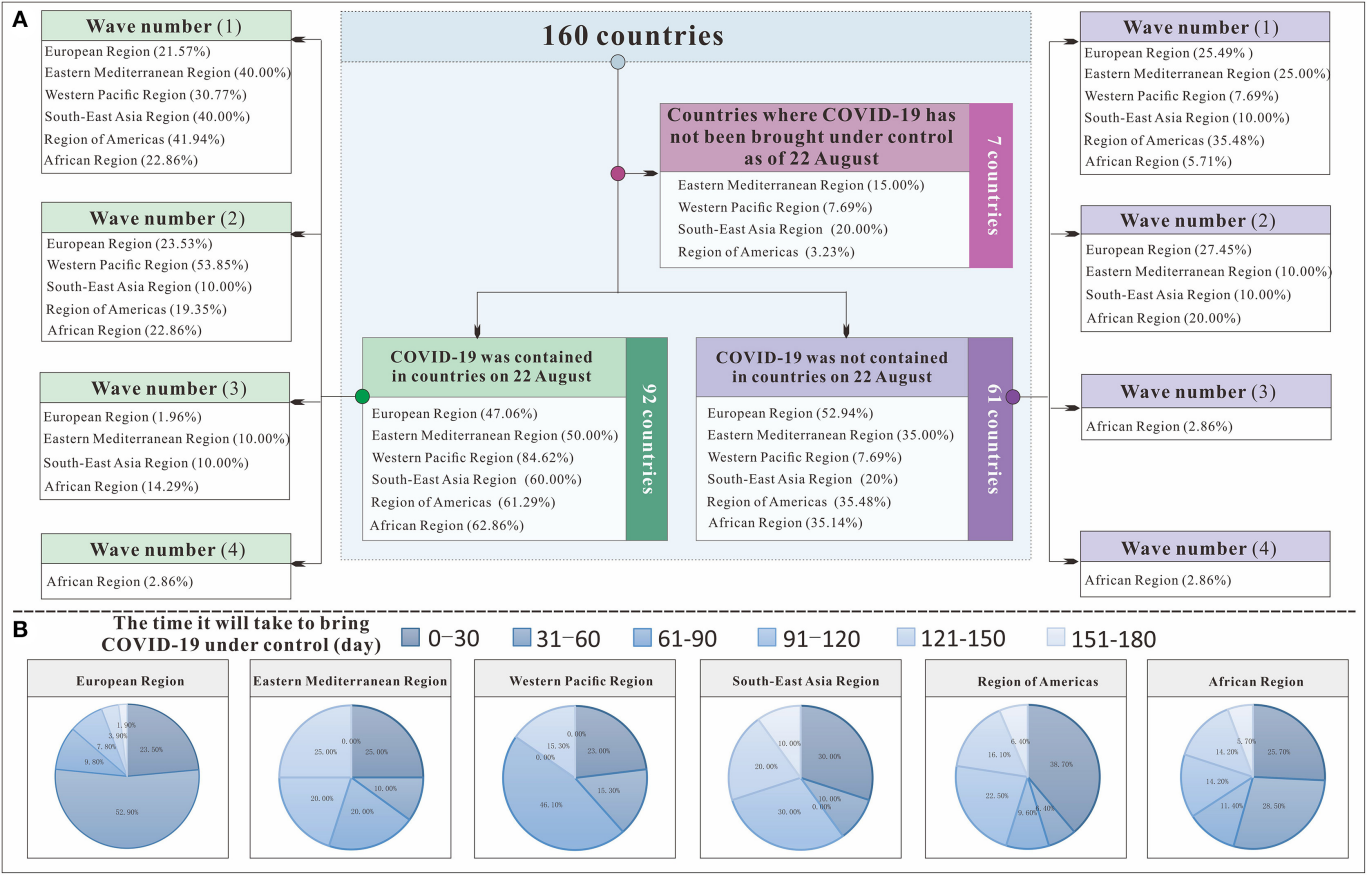
The transmissibility and control effect classification flow chart to control COVID-19 in 160 countries. **(A)** shows the classification process of 160 countries; **(B)** shows the time each region needed to bring COVID-19 under control.

Figure 7A shows that among the 160 countries included in the study, those in the European Region had the strongest national control capability. All 51 European countries included in the study were able to control COVID-19, and the European Region had the highest rate of all the regions (100%), followed by the Region of Americas (85.71%), the Eastern Mediterranean Region (80.95%), the Western Pacific Region (80.87%), the Southeast Asian Region (80%), and the African Region (74.47%). Among all the countries in the European Region, 76.4% were able to control COVID-19 within 60 days. However, in terms of continuous control, the Western Pacific Region did relatively better than the others: 73.76% of its countries on August 22, 2020, at the time of this study. This region was followed by the Region of Americas (52.53%), the Southeast Asian Region (48%), the African Region (46.81%), the European Region (43.63%), and the Eastern Mediterranean Region (40.48%). In countries where the COVID-19 epidemic was continuously controlled, the wave number (*I*_*WN*_) varied among the six regions. In the Western Pacific Region and the Region of Americas, the *I*_*WN*_ was up to 2. Control of COVID-19 was relatively stable. In the European Region, Eastern Mediterranean Region, Southeast Asian Region, and African region, the *I*_*WN*_ was frequently and relatively unstable.

**Figure 8:**
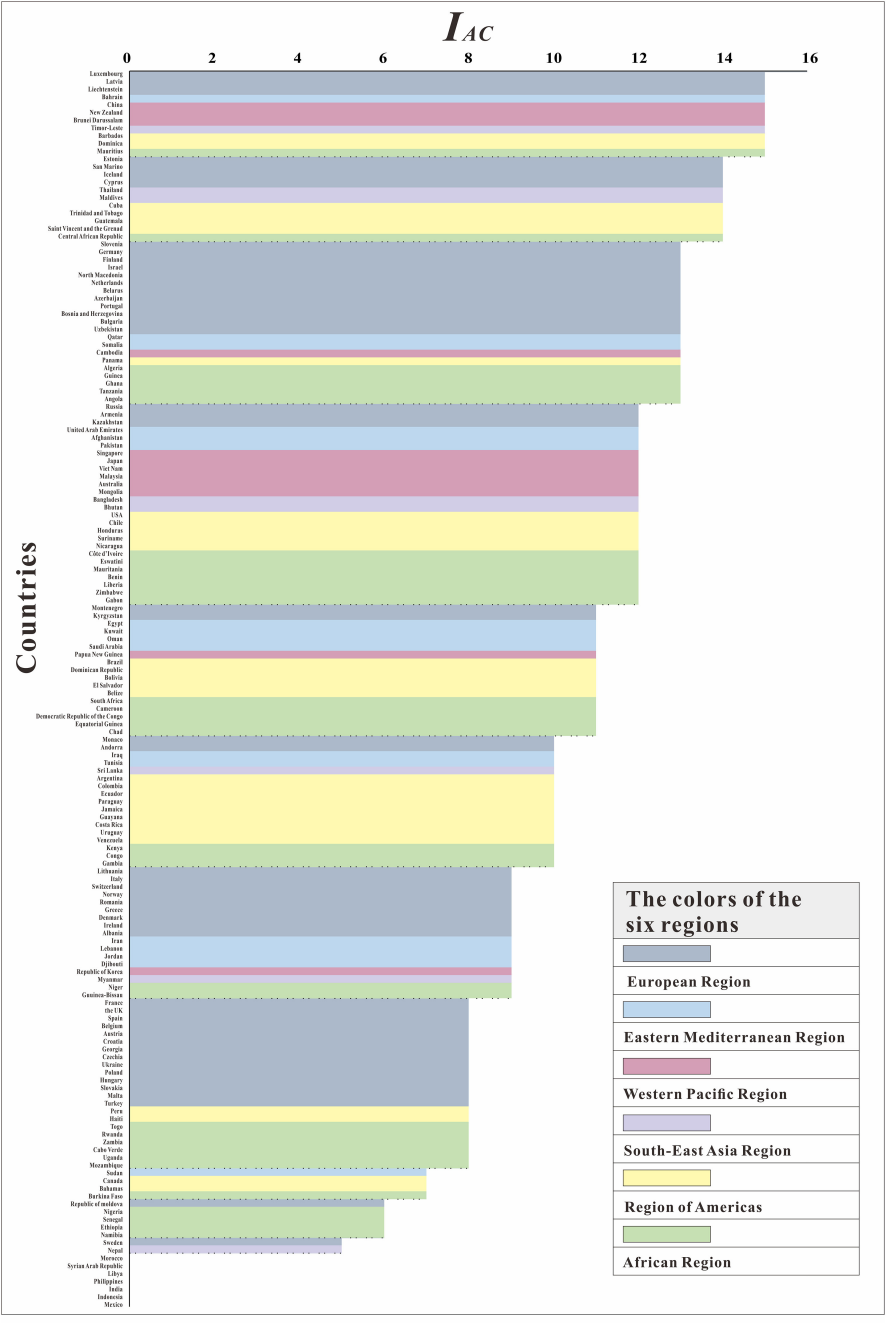
The ranking of *I*_*AC*_ among160 countries.

We further evaluated the ability to control COVID-19 (*I*_*AC*_) in 160 countries. At the most powerful level (*I*_*AC*_ = 15), six regions had countries with control of COVID-19. Although the European Region and the Western Pacific Region each had three countries, in terms of proportion, 5.88% of the European Region and 23.08% of the Western Pacific Region were able to control COVID-19. The Region of Americas had two countries, and the remaining three continents each had one country. Seven countries, including Morocco, Syrian Arab Republic, Libya, Philippines, India, Indonesia, and Mexico, had the worst epidemic control effect (*I*_*AC*_ = 0), and the *R*_*t*_ was <1 for all of these countries throughout the study. From Figure 8, we can see that the countries in the Western Pacific Region were mainly distributed in the front position. Although many countries in the European Region did better, some countries did worse. The Eastern Mediterranean Region and the Southeast Asian Region were relatively evenly distributed in each interval, the countries of the Region of Americas are concentrated at the middle level, and the countries of the African Region are concentrated at the lower level.

### Sensitivity Analysis

**Figure 9:**
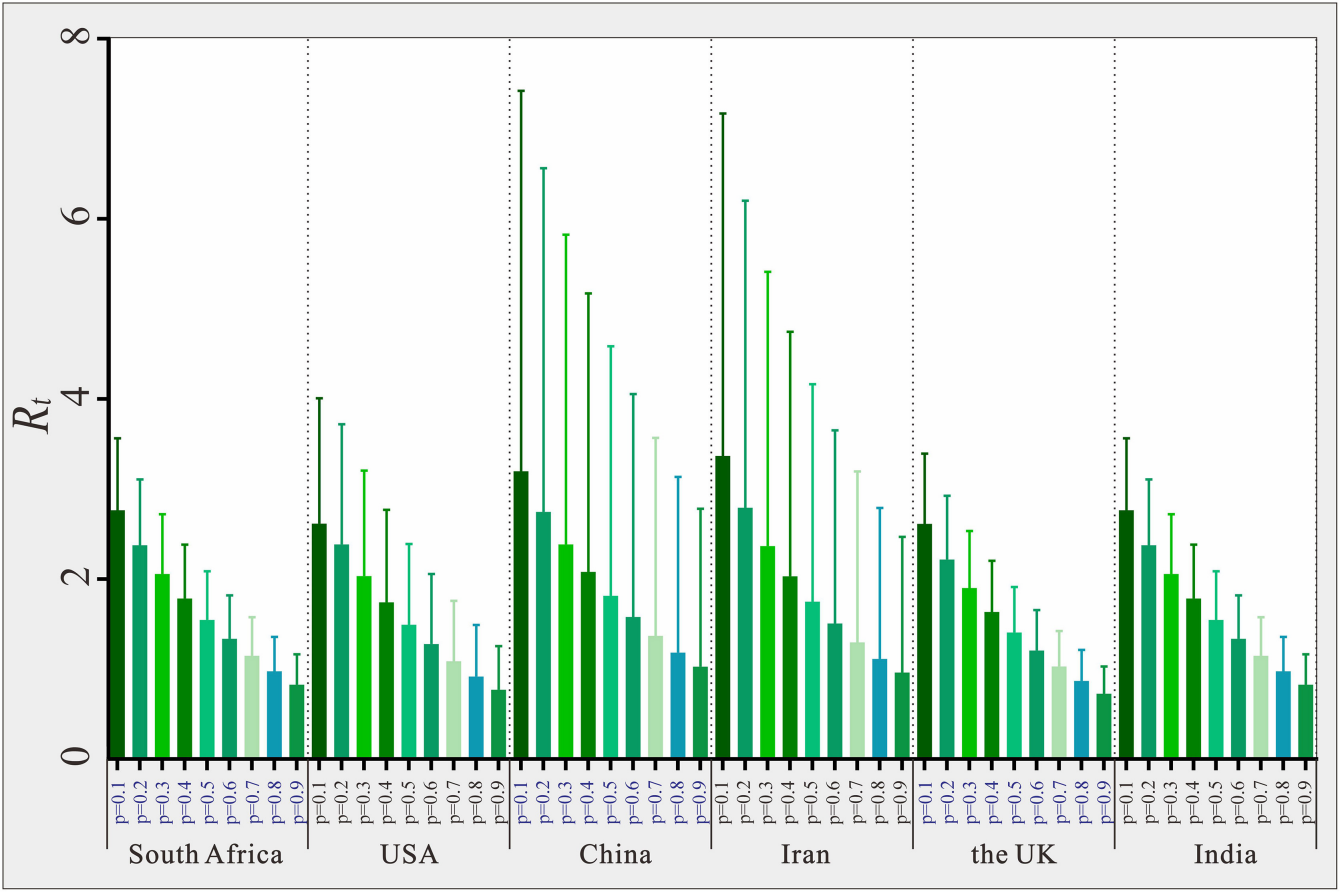
Estimated *R*_*t*_ value based on different *p* in six selected countries in six WHO region.

The six countries randomly selected (South Africa, USA, China, Iran, the UK, and India) located in six WHO regions, respectively. The results of the sensitivity analysis showed that *p* was significantly affected (see Figure 9). The results of sensitivity analysis of countries are consistent in six WHO regions, the larger the value of *p*, the smaller the value of *R*_*t*_. We set the *p* value of each country to 0.2 for each country, which may result in a larger or smaller *R*_*t*_ value deviating from the actual situation. The range of *R*_*t*_ values corresponding to different *p* values in six countries was 1.12–3.45, 1.24–3.97, 4.27–8.78, 5.44–13.77, 4.52–12.19, and 0.99–3.29, respectively. We set the same *p* value in the SEIAR model for all countries to ensure the comparability of results across 160 countries.

## Discussion

We constructed a theoretical epidemiological model, used *R*_*t*_ to reflect the transmissibility of COVID-19 in each country, and explained the differences in the cumulative incidence of COVID-19 across the six regions. Changes in *R*_*t*_ between January 22 and August 22 indicated a slight or moderate effect of local epidemic prevention and control measures. Based on the indicators above, we further constructed the *I*_*AC*_ to evaluate the comprehensive effectiveness of prevention and control measures in 160 countries.

### Model Fitting

From Figure 4, we can see that the model fitted the data of most countries, suggesting that the SEIAR model was useful. Therefore, the SEIAR model that we choose was applicable to the data of these countries. The *R*^2^ of the Southeast Asian Region, the Eastern Mediterranean Region, and the European Region were much higher than those of other regions. This finding may be due to the early outbreak of COVID-19 in these areas, the large number of infected people, or the high detection rate. The reported data from these areas are more in line with the actual data, thus improving the model's fitness. Some countries with low incidences had lower *R*^2^ (e.g., Equatorial Guinea, Guinea, Cameroon, Mongolia, and Liechtenstein). This finding may be related to factors such as the economy of these countries, the level of testing, or the lack of public awareness. There were no obvious epidemiological characteristics in the reported data, which makes poor model fitting results.

### Effect of Prevention and Control Measures Among Six Regions

Coronavirus disease spread regionally in a natural transmission state. However, the estimates of *R*_*t*_ at the beginning of the pandemic may depend on numerous biological, socio-behavioral, and environmental factors (24). The duration and severity of each pandemic phase can vary depending on the characteristics of COVID-19 and the public health response. Italy and Iran were the original hot spots outside China, from which new infections were disseminated to nearby countries. There have been higher magnitudes of COVID-19 infection in Europe and America than in Asia, whereas Europe and America show similar strengths of infection (25). The prevention and control measures adopted and implemented by local governments of various countries in the early stages helped to a certain extent (26, 27). Globally, the number of new cases per day tends to be flat. However, there were still some countries without any reduction in transmissibility after interventions, which suggests that prevention and control measures may not have played important roles in the early stages of the pandemic. Such containment is unlikely to be achievable in most countries around the world, although China succeeded in containing the spread of the pandemic for 2 months by carrying out timely and effective measures (28). Some of the literature has shown that without non-pharmaceutical interventions (including intensive case and contact tracking, isolation of moderately ill patients in containment centers, social distancing, and shutting down public life of a whole province and many major cities outside Hubei), the COVID-19 cases in China would likely have shown a 67-fold increase (interquartile range from 44 to 94) by February 29, 2020 (29). However, it is worth noting that the intervention effect of the African Region was not as good as that of other regions because of its weak healthcare system, which urgently needs to be improved.

The lack of effective interventions is a major driver of the pandemic. Moreover, the fact that the virus is an unknown, new mutant type may be a major factor for the current overwhelming increase in cases despite the preemptively implemented precautions and countermeasures taken by at-risk countries (6). In general, although countries in the Western Pacific region may not be able to control COVID-19 as well as those in the European Region, countries in the European Region have relatively weak continuous response and prevention and control, which is also one of the reasons for the repeated severe epidemics.

Of note, there are some limitations in our study, such as setting of *p* value as 0.2 in 160 countries, but we set the *p* value in the SEIAR model to the same value to ensure comparability among 160 countries. In practice, some asymptomatic patients may die because of the COVID-19 infection. This issue was not considered in our mathematical model and may probably affect the results slightly. However, it would not have a significant impact on our results considering that the mortality of asymptomatic COVID-19 infection was much lower than that of symptomatic infection.

## Data Availability Statement

The original contributions presented in the study are included in the article/Supplementary Material, further inquiries can be directed to the corresponding author/s.

## Author Contributions

GH, TC, JR, and YN made substantial contributions to conception and design. QW, ZC, FX, WZha, ZZ, SL, and YW collected the data. JR, YN, YZ, MY, JX, XL, and WZhe conceived the experiments. JR, FX, QW, KC, YX, LX, SZ, TJ, BZ, YS, and TS conducted the experiments and analyzed the results. JR and QW wrote the manuscript. TC, GH, BZ, and YS revised it critically for important intellectual content. All authors approved the final manuscript and agreed to be accountable for all aspects of the work.

## Conflict of Interest

The authors declare that the research was conducted in the absence of any commercial or financial relationships that could be construed as a potential conflict of interest.

## Publisher's Note

All claims expressed in this article are solely those of the authors and do not necessarily represent those of their affiliated organizations, or those of the publisher, the editors and the reviewers. Any product that may be evaluated in this article, or claim that may be made by its manufacturer, is not guaranteed or endorsed by the publisher.
